# Experimental Study of a Pack of Supercapacitors Used in Electric Vehicles

**DOI:** 10.1155/2017/6702838

**Published:** 2017-08-14

**Authors:** Amari Mansour, Chabchoub Mohamed Hedi, Bacha Faouzi

**Affiliations:** ^1^Laboratory of Computer Science for Industrial Systems, INSAT, Tunis, Tunisia; ^2^Computer Embedded System (CES) Laboratory, National School of Engineers of Sfax (ENIS), Sfax, Tunisia

## Abstract

Electric vehicles have recently attracted research interest. An electric vehicle is composed of two energy sources, such as fuel cells and ultracapacitors, which are employed to provide, respectively, the steady-state and transient power demanded by the vehicle. A bidirectional DC-DC converter is needed to interface the ultracapacitor to a DC bus. The pack of ultracapacitor consists of many cells in series and possibly also in parallel. In this regard, this paper introduces a comparative study between two packs of supercapacitors. The first supercapacitor pack is composed of ten cells in series but the second supercapacitor pack is composed of five cells in series and two parallel circuits. Each cell is characterized by 2.5 V and 100 F. A number of practical tests are presented.

## 1. Introduction

**Figure 1:**
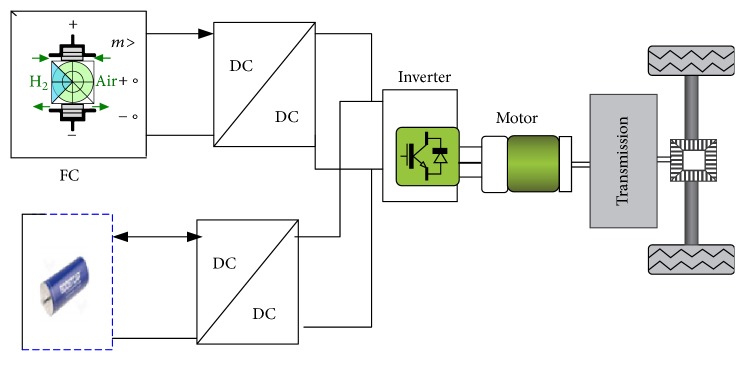
Electric vehicle system.

Rapidly increasing population, energy consumption, and the need to reduce emissions through the conventional vehicle have motivated researchers to study the electric hybrid vehicles (EHVs) [1]. Usually, the electric hybrid vehicles architecture includes two or more energy sources with their associated energy converters as shown in Figure 1.

**Figure 2:**
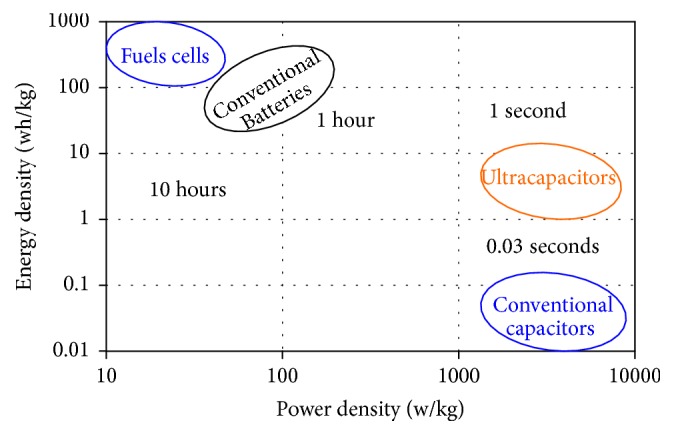
Ragone diagram of energy storage sources.

The main source is a fuel cell, with high energy storage capability; it is the electrochemical devices that convert chemical energy of a reaction directly into electrical energy [2]. This has slow dynamic to response under load variation and does not allow the recuperation of energy from the load [3]. The second source is the storage system; it produces the lacking power in acceleration and absorbs excess power in braking function. Batteries and ultracapacitors are employed as energy storage system in many hybrid applications. Recently, ultracapacitors have been explored better than batteries in the electrical vehicles because they present considerably higher power densities than those of batteries, and extremely higher energy densities than those of conventional electrolytic capacitors [4, 5]. The graph in Figure 2 illustrates the regions of applicability of the various energy storage systems [6].

Other than electric vehicles, supercapacitor can also be used as additional energy storage for hybrid wind and photovoltaic system. It charges energy when it is windy or sunny and discharges when there is no power generated from photovoltaic or wind due to the sudden passing clouds disturbance or very low wind speed [7]. Hence, it is necessary to understand the characteristics of the supercapacitor and determine these different electric models. In the literature many models have been developed such as electrochemical models and equivalent circuit ones. This paper presents a practical comparative study of equivalent circuit models of ultracapacitors used in electric vehicles. This paper is summarized as follows.  Section 2 describes the ultracapacitor model.  Section 3 details the topology of the boost, the operation mode, and the average model.  Section 4 evaluates the simulation and the experimental results. And finally the conclusion is presented in Section 5.

## 2. Ultracapacitor Modeling

Ultracapacitors consist of two electrodes and an ion-permeable separator that prevents physical contact between the two electrodes [8]. They are characterized by high power density, high energy efficiency, low internal resistance, long cycle life, and fast charging/discharging time. In recent years as ultracapacitors become used more widely, several different circuit models have been proposed in the literature [9].

### 2.1. RC Circuit Model

**Figure 3:**
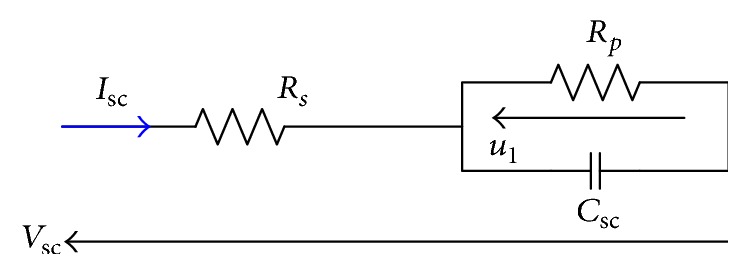
Classical model.

The circuit schematic in Figure 3 represents the simple RC model for a ultracapacitor. It is comprised of three ideal circuit elements: a series resistor *R*_*s*_: it is called the equivalent series resistor (ESR) and contributes to the energy loss component of the ultracapacitor during charging or discharging; a parallel resistor *R*_*p*_: it is called the leakage resistance, and a capacitance *C*_sc_. This model is developed and validated experimentally in many works [5, 10–12].

The dynamic can be described as

(1) du1tdt=−u1tRpCsc+IscCscVsct=RsIsc+u1t.

**Figure 4:**
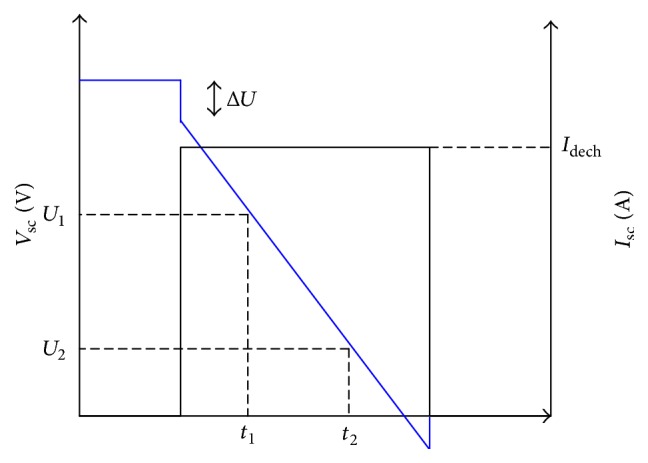
Evolution of voltage discharged with a constant current.

The ultracapacitor is discharged with a constant current and the result is presented in Figure 4.

The equivalent series resistor is obtained through the following equation:

(2) $$\begin{array}{c} R_{s}=\frac{\Delta U}{I_{dech}}, \end{array}$$

where Δ*U* and *I*_dech_ denote, respectively, the voltage drop which is observed at the beginning of the discharge and the discharge current.

The capacitor can be expressed by

(3) $$\begin{array}{c} C_{sc}=\frac{I_{dech}\left(t_{2}-t_{1}\right)}{U_{1}-U_{2}}. \end{array}$$

The parallel resistor is calculated using

(4) $$\begin{array}{c} R_{p}=\frac{U_{1}}{I_{L}}, \end{array}$$

where *U*_1_ and *I*_*L*_ denote, respectively, the open circuit voltage and the leakage current.

### 2.2. Three-Stage Ladder Model

**Figure 5:**
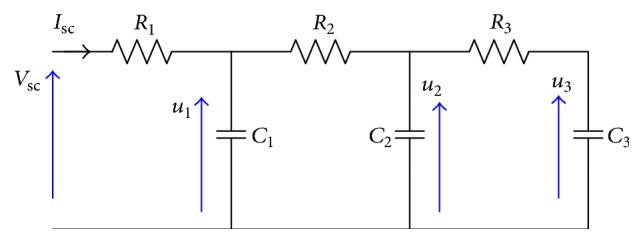
Three-stage ladder model.

The three-stage ladder model is shown in Figure 5. It is composed of three resistors and three capacitors. This model is developed in many references [13–15].

To model the three-stage ladder model we choose three state variables including capacitors voltages *u*_1_, *u*_2_, and *u*_3_. The state space representation is described by the following equation:

(5) dxdt=Ax+Buy=Cx+Du,

where $x={\left[\begin{array}{ccc} u_{1} & u_{2} & u_{3} \end{array}\right]}^{T}$, *u* = *I*_sc_, and *y* = *V*_sc_.

According to basic electrical circuit principles, the matrix expressions are the following:

(6) A=−1R2C11R2C10−1R2C2−R2+R3R2R3C21R3C201R3C3−1R3C3;B=1C100T,C=100,D=Rsc.

### 2.3. The Dynamic Model

**Figure 6:**
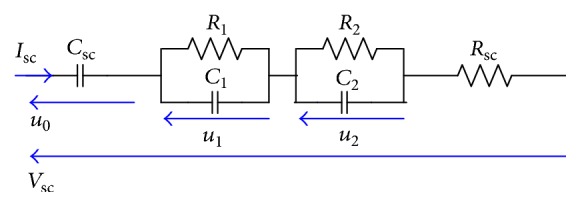
The dynamic model.

In this case, the equivalent circuit is composed of two parallel circuit RC. It has the structure shown in Figure 6 where *u*_0_ denotes the voltage across the bulk capacitor *C*_sc_, *u*_1_ and *u*_2_ denote the voltages of the two RC circuits, respectively, *V*_sc_ denotes the output voltage, and *R*_sc_ denotes the series resistance [16].

The state space representation can be derived as

(7) du0 dtdu1dtdu2dt=0000−R1C1000−R2C2u0u1u2+1Csc1C11C2IscVsc=u0+u1+u2+RscIsc.

## 3. Topology of Converter

**Figure 7:**
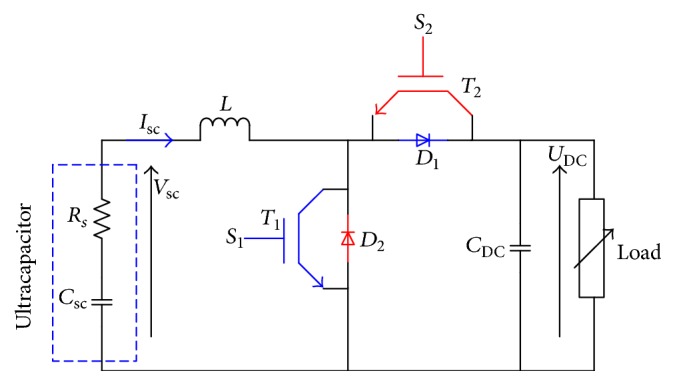
Topology of DC-DC converter.

There are many possible topologies of DC-DC converters which interface the ultracapacitor and the DC link. The DC-DC converters can be divided into two categories depending on using the galvanic insulation or not: nonisolated converter or isolated converter. The proposed converter has two basic operating modes: the buck and the boost mode. In this context, many papers have been elaborated [17–19].  Figure 7 shows the converter topology. The chosen DC-DC converter is composed of two IGBTs, two diodes, inductor, and capacitor.

There are two modes. (a) Charging mode: in this case, the load delivers the energy to the capacitor through the IGBT (*T*_2_), the diode (*D*_2_), and the inductor *L*. (b) Discharging mode: in this case, the ultracapacitor delivers the energy to the load through the IGBT (*T*_1_), the diode *D*_1_, and the inductor *L*.  Table 1 shows the state of any switch for two modes, where *T*_*s*_, *d*_1_,and *d*_2_ denote, respectively, the switching period, the duty cycle in charging mode, and the duty cycle in discharging mode.

The key factors that determine the parameters of the circuit include the limit ripple of the ultracapacitor current (*δ*(*I*_sc_)) and the ripple of the output voltage (*δ*(*V*_DC_)), the switching period (*T*_*s*_), and the output power (*P*_out_). In discharging mode the inductor and the output capacitor are described by the following equation:

(8) L≥Vscd2TsIscδIscC≥d21−d22TsIscVscδVDC.

In this application, the ultracapacitor voltage is 25 V, if we choose *I*_sc_ = 4 A, *V*_DC_ = 125 V, *T*_*s*_ = 5*e*^−4^ s, and *δ*(*I*_sc_) = *δ*(*V*_DC_) = 5%. The boost parameters are *d*_1_ = 0.8, *L* = 50 mH, and *C* = 256 *μ*F.

To model the boost converter, we used the average method. In this case, it is assumed that all components are ideal; that is, there is no internal resistance in the circuit and the circuit components do not consume any energy. We choose two state variables including output voltage and inductor current. The system state space representation is

(9) x.=Ax+B·uy=C·x,

where *u* is the vector of inputs, *y* is the outputs, and *x* is the status variables vector.

(10) x=isct,vDCtT,yt=vDCt,ut=vsct.

During the switching period, the topology converter can be divided into two equivalent circuits.

**Case 1 ($t\in \left[\begin{array}{cc} 0 & d_{1}T_{s} \end{array}\right]$). **

**Figure 8:**
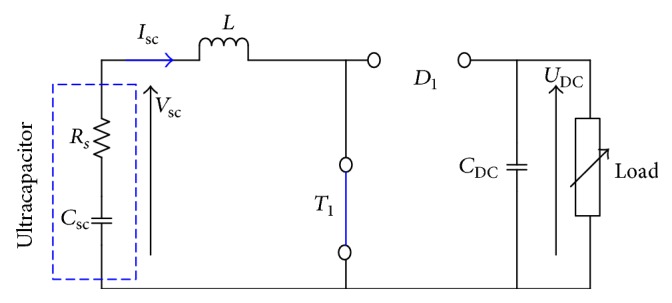
Equivalent circuit for Case 1.

The switch *T*_1_ is ON; the equivalent circuit can be simplified as shown Figure 8.

We can write the state space by the following equation:

(11) x.=A1x+B1·uy=C1·x.

The matrices *A*_1_, *B*_1_, and *C*_1_ can be expressed as follows:

(12) A1=000−1RLoadCDC,B1=1L0,C1=01.

**Case 2 ($t\in \left[\begin{array}{cc} d_{1}T_{s} & T_{s} \end{array}\right]$). **

**Figure 9:**
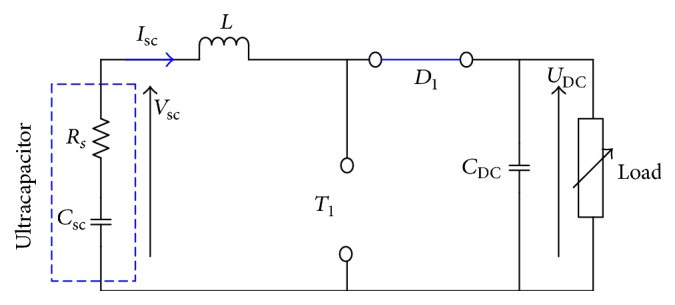
Equivalent circuit for Case 2.

In this case, the switch *T*_1_ is OFF; the ultracapacitor delivers the energy to the load through the inductor and the diode *D*_1_. The equivalent circuit can be simplified as shown in Figure 9.

Using Kirchhoff law, the state space model is the following:

(13) x.=A2x+B2·uy=C2·x.

The matrices *A*_2_, *B*_2_, and *C*_2_ can be expressed as follows:

(14) A2=001CDC−1RLoadCDC,B2=1L0,C2=01.

The average state model is

(15) x.=Ax+B·uy=C·x,

where

(16) A=d1·A1+1−d1A2B=d1B1+1−d1B2C=d1C1+1−d1C2.

If we insert (12) and (14) in (16), the matrices *A*, *B*, and *C* are

(17) A=0d1−1L1−d1CDC−1RLoadCDC,B=1L0,C=01.

**Figure 10:**
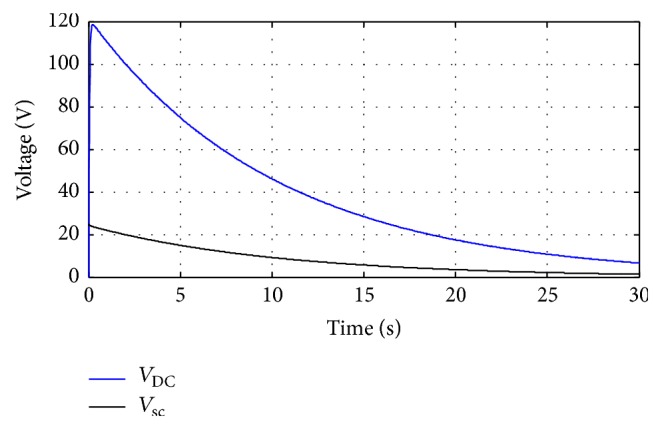
Output and the ultracapacitor voltage.

In MATLAB, we simulate the open loop response to verify the average model using the circuit parameter defined by (8). We initialize the system by choosing the duty cycle at 0.8 and the load resistor at 25 *Ω*.  Figure 10 illustrates the DC link and the ultracapacitor voltage.

## 4. Experimental Validations

**Figure 11:**
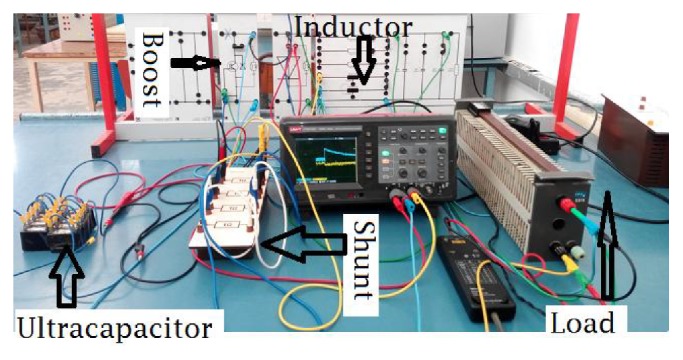
Test circuit.

A test system has been developed allowing the supercapacitor to be charged and discharged and to calculate the equivalent circuit parameters. The test circuit shown in Figure 11 allows delivering a range of voltages and currents (charge and discharge mode) and hence is capable of characterizing the range of supercapacitors used in this application. The prototype consists of ten cells (maximum stack voltage of 25 V), a boost converter (IGBT module, diode, and inductor), and a load.

The used ultracapacitor is Wima (made in Germany). These specifications are given in Table 2.

Two experiments are carried out using this setup. In the first experiment, the ten cells are connected in series, while the second one demonstrates the functionality of the system when the supercapacitors are connected in two parallel circuits.

### 4.1. Charging Mode

#### 4.1.1. Case  1

**Figure 12:**
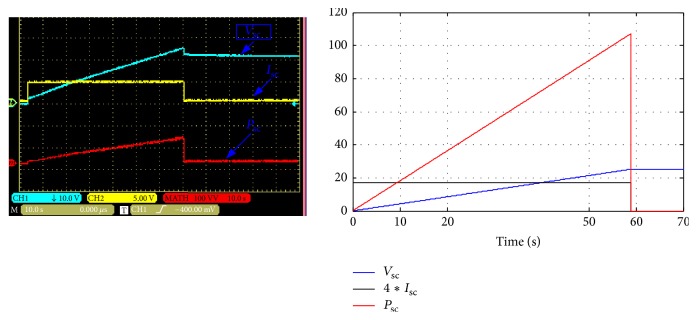
Voltage, current, and power waveforms of the ultracapacitors (charging mode-series).

In this case the ultracapacitors are connected in series and are charging with constant current (4.25 A).  Figure 12 shows the current, voltage, and power waveforms of the ultracapacitor.

Figure 12 shows that the charging time of the supercapacitor is equal to 66 seconds. In this case, the equivalent capacitor of the ultracapacitor module is 10.8 F. The cell capacitor and the equivalent series resistor are equal, respectively, to 108 F and 36 m*Ω*.

The voltage cells obtained from this condition are summarized in Table 3.

According to this table, the voltages of the different cells are not identical; that is why it is important to use a voltage balancing circuit.

#### 4.1.2. Case  2

**Figure 13:**
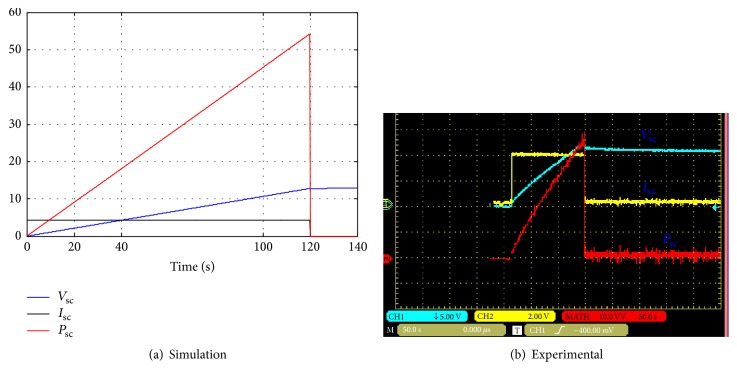
Voltage, current, and power waveforms of the ultracapacitors (charging mode-parallel).

In this case, the ten cells of this module are divided into two parallel circuits and charged with constant current as shown in Figure 13. The charging time is equal to 140 s.

### 4.2. Discharging Mode

Initially, the supercapacitor pack is fully charged. It provides power to the load through the IGBT, smoothing inductance, and diode. The control circuit allows varying the duty cycle and the switching frequency.

#### 4.2.1. Case  1: The Ultracapacitors Cells Are Connected in Series

**Figure 14:**
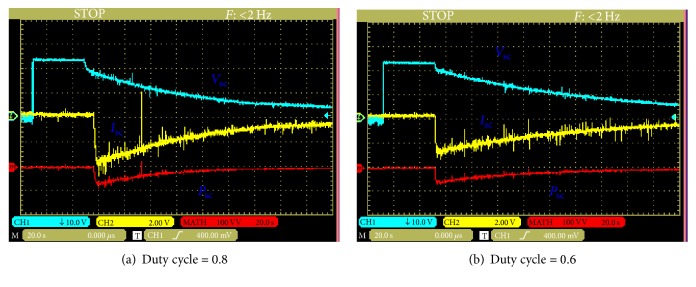
Voltage, current, and power waveforms of the ultracapacitors (discharging mode: series).

The experimental results in discharging mode are obtained with (0.8 and 0.6) as the duty cycle and loads (25 *Ω*).  Figure 14 presents the voltage, current, and power of ultracapacitors for two- duty cycle.

#### 4.2.2. Case  2: The Ultracapacitors Cells Are Connected into Two Parallel Circuits

**Figure 15:**
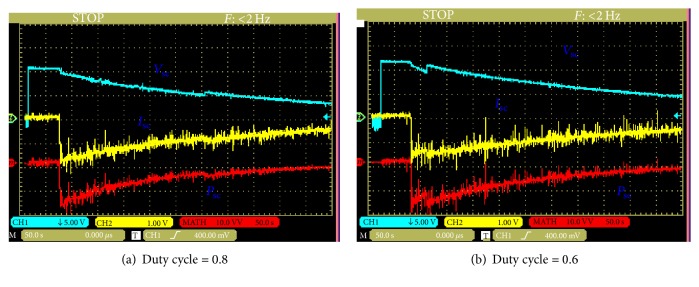
Voltage, current, and power waveforms of the ultracapacitors (discharging mode: parallel).

In this case, the ten cells of this module are divided into two parallel circuits and deliver the energy to the load through the boost and the inductor.  Figure 15 shows the voltage, current, and power for two values of duty cycle (0.8 and 0.6).

**Figure 16:**
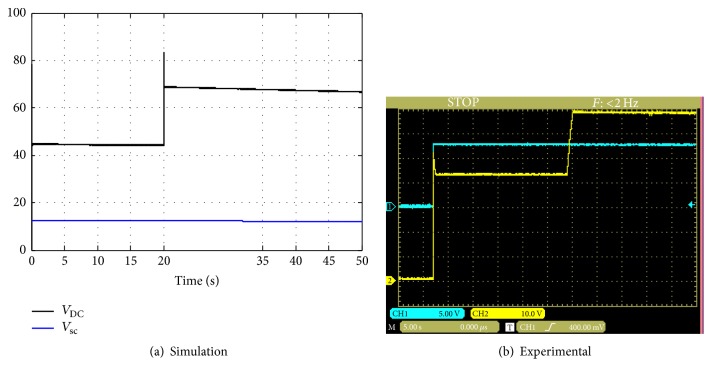
Simulation and experimental results for step change of the duty cycle.

In this case the current system and average model are simulated with *d*_1_ = 0.72 and then in time of 20 seconds the duty cycle changes from 0.72 to 0.82.  Figure 16 shows the simulation and the experimental results.

## 5. Conclusion

An ultracapacitor bank has been simulated and tested. The system uses a IGBT boost converter; the control of the system measures the ultracapacitor voltage, the ultracapacitor current, and the output voltage. A simple experimental methodology is proposed for a systematic validation of average models in time domain and tested the ultracapacitor performance for two connections (series and parallel). We have assumed a purely resistive load, which is not always the case in practice (acceleration and regenerative braking for an electric vehicle). It can be interesting to study the system (buck-boost + ultracapacitor) associated with an electric machine.

## Figures and Tables

**Table 1:** State of the switches.

Switch	Charging mode	Discharging mode
*T*_1_	0	$\left[\begin{array}{cc} 0 & d_{1}T_{s} \end{array}\right]$
*T*_2_	$\left[\begin{array}{cc} 0 & d_{2}T_{s} \end{array}\right]$	0
*D*_1_	0	$\left[\begin{array}{cc} 0 & d_{1}T_{s} \end{array}\right]$
*D*_2_	$\left[\begin{array}{cc} d_{2}T_{s} & T_{s} \end{array}\right]$	0

**Table 2:** Ultracapacitor specifications.

Parameter	Value
Voltage cell	*V*_sc_ = 2.5 V
Capacitor cell	*C*_sc_ = 100 F
Resistor	Resr = 36 mΩ
Cell number	10

**Table 3:** Voltage cells value.

Number of cells	Cell voltage (V)
1	2.603
2	2.609
3	2.3
4	2.571
5	2.589
6	2.538
7	2.482
8	2.484
9	2.512
10	2.451
